# Supplementary material for: Corona virus fear among health workers during the early phase of pandemic response in Nepal: A web-based cross-sectional study
Source: PLOS Glob Public Health. 2021 Dec 15;1(12):e0000083. doi: 10.1371/journal.pgph.0000083 (PMC10022105; doi:10.1371/journal.pgph.0000083)
Supplement: S1 Table — (DOCX) [file pgph.0000083.s001.docx]

**S1 Table: Descriptive analysis of the items of the English version FCV-19S**

| **Item** | **Mean (SD)** | **Skewness** | **Kurtosis** | **Corrected item-total correlation** | **Cronbach’s alpha if deleted** |
| --- | --- | --- | --- | --- | --- |
| FCV-19 S1 | 2.73 (1.11) | .167 | -.791 | .656 | .840 |
| FCV-19 S2 | 2.60 (1.12) | .219 | -1.066 | .679 | .836 |
| FCV-19 S2 | 2.03 (1.00) | .835 | .151 | .634 | .843 |
| FCV-19 S4 | 2.59 (0.87) | 1.176 | .075 | .629 | .844 |
| FCV-19 S5 | 2.69 (1.20) | .149 | -1.131 | .674 | .838 |
| FCV-19 S6 | 2.30 (0.63) | 2.118 | 3.839 | .542 | .858 |
| FCV-19 S7 | 2.13 (1.04) | .818 | .033 | .664 | .838 |

Note: Cronbach’s alpha of overall scale: 0.862
