# Supplementary material for: Corona virus fear among health workers during the early phase of pandemic response in Nepal: A web-based cross-sectional study
Source: PLOS Glob Public Health. 2021 Dec 15;1(12):e0000083. doi: 10.1371/journal.pgph.0000083 (PMC10022105; doi:10.1371/journal.pgph.0000083)
Supplement: S2 Table — (DOCX) [file pgph.0000083.s002.docx]

**S2 Table: Correlation of FCV-19 S with HADS-A, HADS-D and ISI (N=475)**

| **Scale** | **Correlation** | **HADS-A** | **HADS-D** | **ISI** |
| --- | --- | --- | --- | --- |
| FCV-19 S | Pearson Correlation Coefficient | 0.513 | 0.425 | 0.367 |
|  | P value | <0.001 | <0.001 | <0.001 |
| FCV-19 S1 | Pearson Correlation Coefficient | 0.374 | 0.302 | 0.200 |
|  | P value | <0.001 | <0.001 | <0.001 |
| FCV-19 S2 | Pearson Correlation Coefficient | 0.373 | 0.302 | 0.213 |
|  | P value | <0.001 | <0.001 | <0.001 |
| FCV-19 S3 | Pearson Correlation Coefficient | 0.326 | 0.336 | 0.243 |
|  | P value | <0.001 | <0.001 | <0.001 |
| FCV-19 S4 | Pearson Correlation Coefficient | 0.413 | 0.287 | 0.320 |
|  | P value | <0.001 | <0.001 | <0.001 |
| FCV-19 S5 | Pearson Correlation Coefficient | 0.410 | 0.314 | 0.283 |
|  | P value | <0.001 | <0.001 | <0.001 |
| FCV-19 S6 | Pearson Correlation Coefficient | 0.380 | 0.370 | 0.433 |
|  | P value | <0.001 | <0.001 | <0.001 |
| FCV-19 S7 | Pearson Correlation Coefficient | 0.422 | 0.343 | 0.315 |
|  | P value | <0.001 | <0.001 | <0.001 |
