# Supplementary material for: Corona virus fear among health workers during the early phase of pandemic response in Nepal: A web-based cross-sectional study
Source: PLOS Glob Public Health. 2021 Dec 15;1(12):e0000083. doi: 10.1371/journal.pgph.0000083 (PMC10022105; doi:10.1371/journal.pgph.0000083)
Supplement: S3 Table — (DOCX) [file pgph.0000083.s003.docx]

**S3 Table: Fear of COVID-19 and its associated factors**

| **Variables** | **Categories** | **Absence of COVID fear (n=256)**  **N (%)** | **Presence of COVID fear (n=219)**  **N (%)** | **P value** | **Mean (SD)** | **P value** |
| --- | --- | --- | --- | --- | --- | --- |
| Gender |  |  |  |  |  |  |
|  | Male | 142 (63.1) | 83 (36.9) | <0.001 | 16.02 (4.86) | <0.001 |
|  | Female | 114 (45.6) | 136 (54.4) |  | 18.01 (5.35) |  |
| Education |  |  |  |  |  |  |
|  | Technical SLC and intermediate | 41 (43.6) | 53 (56.4) | 0.020 | 18.73 (5.84) | 0.002 |
|  | Bachelors | 149 (53.8) | 128 (46.2) |  | 16.81 (4.87) |  |
|  | Masters and above | 66 (63.5) | 38 (36.5) |  | 16.26 (5.23) |  |
| Marital status |  |  |  |  |  |  |
|  | Single | 168 (56.2) | 131 (43.8) | 0.191 | 16.72 (4.94) | 0.060 |
|  | Ever married | 88 (50.0) | 88 (50.0) |  | 17.65 (5.62) |  |
| Family type |  |  |  |  |  |  |
|  | Nuclear | 165 (53.7) | 142 (46.3) | 0.999 | 17.06 (5.25) | 0.974 |
|  | Joint and extended | 90 (53.9) | 77 (46.1) |  | 17.08 (5.15) |  |
| Living with child |  |  |  |  |  |  |
|  | Yes | 58 (48.7) | 61 (51.3) | 0.193 | 17.88 (5.55) | 0.049 |
|  | No | 198 (55.6) | 158 (44.4) |  | 16.79 (5.08) |  |
| Living with elderly |  |  |  |  |  |  |
|  | Yes | 80 (49.1) | 83 (50.9) | 0.128 | 17.62 (5.47) | 0.095 |
|  | No | 176 (56.4) | 136 (43.6) |  | 16.78 (5.06) |  |
| Work position |  |  |  |  |  |  |
|  | Front line worker | 123 (57.5) | 91 (42.5) | 0.156 | 17.00 (5.39) | 0.813 |
|  | Second line worker | 133 (51.0) | 128 (49.0) |  | 17.11 (5.07) |  |
| Type of health facility |  |  |  |  |  |  |
|  | Primary | 48 (57.1) | 36 (42.9) | 0.510 | 17.23 (5.36) | 0.956 |
|  | Secondary and tertiary | 208 (53.2) | 183 (46.8) |  | 17.03 (5.19) |  |
| Affected district |  |  |  |  |  |  |
|  | Yes | 176 (50.3) | 174 (49.7) | 0.008 | 17.37 (5.26) | 0.032 |
|  | No | 80 (64.0) | 45 (36.0) |  | 16.21 (5.00) |  |
| Profession |  |  |  |  |  |  |
|  | Doctor | 107 (66.0) | 55 (34.0) | <0.001 | 15.89 (4.52) | <0.001 |
|  | Nurse | 61 (36.5) | 106 (63.5) |  | 19.00 (5.34) |  |
|  | Others | 88 (60.3) | 58 (39.7) |  | 16.17 (5.19) |  |
| Age (years) |  |  |  |  |  |  |
|  | 20-40 | 247 (54.5) | 206 (45.5) | 0.211 | 16.99 (5.21) | 0.137 |
|  | >40 | 9 (41.0) | 13 (59.0) |  | 18.68 (5.19) |  |
| Ethnicity |  |  |  |  |  |  |
|  | Brahmin/Chhetri | 184 (58.8) | 129 (41.2) | 0.004 | 16.49 (5.06) | 0.001 |
|  | Janjati | 48 (41.4) | 68 (58.6) |  | 18.41 (5.07) |  |
|  | Madhesi | 18 (62.1) | 11 (37.9) |  | 16.48 (6.23) |  |
|  | Others | 6 (35.3) | 11 (64.7) |  | 19.65 (5.05) |  |
| Precautionary measure in workplace |  |  |  |  |  |  |
|  | Not sufficient | 193 (51.5) | 182 (48.5) | 0.040 | 17.53 (5.03) | <0.001 |
|  | Sufficient | 63 (63.0) | 37 (37.0) |  | 15.35 (5.53) |  |
| Family member with chronic conditions |  |  |  |  |  |  |
|  | Yes | 127 (49.0) | 132 (51.0) | 0.020 | 17.66 (5.26) | 0.007 |
|  | No | 129 (59.7) | 87 (41.3) |  | 16.36 (5.08) |  |
| Work experience |  |  |  |  |  |  |
|  | Up to 5 year | 188 (56.0) | 148 (44.0) | 0.162 | 16.91 (5.110 | 0.300 |
|  | >5 years | 68 (48.9) | 71 (51.1) |  | 17.45 (5.45) |  |
| Faced stigma |  |  |  |  |  |  |
|  | Yes | 119 (46.7) | 136 (53.3) | 0.003 | 18.03 (5.31) | <0.001 |
|  | No^a^ | 137 (62.3) | 83 (37.7) |  | 15.95 (4.88) |  |
| Aware of government incentive |  |  |  |  |  |  |
|  | Yes | 158 (58.5) | 112 (41.5) | 0.020 | 16.67 (5.14) | 0.054 |
|  | No | 98 (47.8) | 107 (52.2) |  | 17.60 (5.28) |  |
| Working overtime |  |  |  |  |  |  |
|  | Yes | 122 (52.4) | 111 (47.6) | 0.510 | 17.23(5.50)) | 0.512 |
|  | No | 134 (55.4) | 108 (44.6) |  | 16.91 (4.93) |  |
| History of medication |  |  |  |  |  |  |
|  | Yes | 15 (68.2) | 7 (31.8) | 0.169 | 16.50 (5.42) | 0.602 |
|  | No | 241 (53.2) | 212 (46.8) |  | 17.09 (5.21) |  |

^a^No and do not want to answer merged as No
