# Supplementary material for: Corona virus fear among health workers during the early phase of pandemic response in Nepal: A web-based cross-sectional study
Source: PLOS Glob Public Health. 2021 Dec 15;1(12):e0000083. doi: 10.1371/journal.pgph.0000083 (PMC10022105; doi:10.1371/journal.pgph.0000083)
Supplement: Attachment — Submitted filename: Reviwers reply_fear.docx [file pgph.0000083.s004.docx]

Nov 1, 2021

To The Editor

PLOS Global Public Health

Re: [PGPH-D-21-00428] “Corona virus fear among health workers during the early phase of pandemic response in Nepal: a web based cross sectional study”

Dear Editor,

Thank you so much for your and reviewer’s comments on our manuscript. We have carefully revised the manuscript in response to extensive and insightful comments we received. We are pleased that there is interest in the topic and are grateful for the constructive comments.

We have addressed the comments point by point and have made corresponding changes in the manuscript in track changes. Appended below please find the list of all reviewers and editors comments along with our response to each point.

We hope this revised version will be suitable for the publication in PLOS Global Public Health.

Yours sincerely,

Kiran Paudel

[Kiranpaudel59@gmail.com](mailto:Kiranpaudel59@gmail.com)

Corresponding author.

#Editors’ reply

1. In your Methods section, please provide additional information about the participant recruitment method and the demographic details of your participants. Please ensure you have provided sufficient details to replicate the analyses such as: 
a) a description of any inclusion/exclusion criteria that were applied to participant recruitment, 
b) a statement as to whether your sample can be considered representative of a larger population, and 
c) a more detailed description of how participants were recruited.

Reply: Thank you for the suggestion. Details about the recruitment and the demographic details of the study participants have been mentioned in an article published elsewhere. Link to the article is <https://globalizationandhealth.biomedcentral.com/articles/10.1186/s12992-020-00621-z>

2. During our internal evaluation of the manuscript, we found text overlap between your submission and the following previously published works:

<https://www.grin.com/document/1007212>

Please revise the manuscript to rephrase the duplicated text and cite your sources.

Reply: Thank you for the note. As we went through the link, we found that the author is a doctoral thesis student and had cited our work (reference number of the work: 24) which was on pre-print platform during his literature review. Thus, no revision was made in this regard.

3. Thank you for providing consent information for your study. However, we note that you have not provided ethical approval information. We understand that the framework for ethical oversight requirements for studies of this type may differ depending on the setting and we would appreciate some further clarification regarding your research. Could you please provide further details on why your study is exempt from the need for approval and confirmation from your institutional review board or research ethics committee (e.g., in the form of a letter or email correspondence) that ethics review was not necessary for this study? Please include a copy of the correspondence as an "Other" file.

Reply: Ethical approval for the study was obtained from the Ethical Review Board of Nepal Health Research Council (Reference number: 2192, 315/2020). We have already included this information in our manuscript in the study design, participants, and procedures sub-section of the methods section.

4. In the online submission form, you indicated that "Data can be make available after asking with first and corresponding author in reasonable request.". All PLOS journals now require all data underlying the findings described in their manuscript to be freely available to other researchers, either 1. In a public repository, 2. Within the manuscript itself, or 3. Uploaded as supplementary information.
This policy applies to all data except where public deposition would breach compliance with the protocol approved by your research ethics board. If your data cannot be made publicly available for ethical or legal reasons (e.g., public availability would compromise patient privacy), please explain your reasons by return email and your exemption request will be escalated to the editor for approval. Your exemption request will be handled independently and will not hold up the peer review process, but will need to be resolved should your manuscript be accepted for publication. One of the Editorial team will then be in touch if there are any issues.

Reply: Thank you for flagging the importance of the public data repository. We will make the data publically available and data will be uploaded as a supplementary information in the form of tables.

5. We have noticed that you have uploaded supporting information but you have not included a list of legends.  Please add a full list of legends for all supporting information files (including figures, table and data files) after the references list.

Reply: Thank you for the suggestion. Now, we have added full list of legends for all supporting information after the references list in the main manuscript.

6. Please review your reference list to ensure that it is complete and correct. If you have cited papers that have been retracted, please include the rationale for doing so in the manuscript text, or remove these references and replace them with relevant current references. Any changes to the reference list should be mentioned in the rebuttal letter that accompanies your revised manuscript. If you need to cite a retracted article, indicate the article’s retracted status in the References list and also include a citation and full reference for the retraction notice.

Reply: We have reviewed all the references and have made sure that the relevant references are only included.

The following changes have been made

For the reference 15 and 25, we have cited published articles as the previously cited papers were pre-prints.

**#Reviewer_Reply**

Reviewer#1:

The topic seems to be relevant with the current pandemic situation. It may be helpful for policy implication and reference for future research. However, abstract and introduction seems to have little information. Could you please add some references for this article? Other factors remain excellent.

Reply: Thank you for the suggestion. We now have added some health system related information of Nepal in the introduction section which are relevant with the current pandemic situation.

Reviewer #2:

Perhaps consider bulking up your introduction with additional Nepal-specific information: have there been anything else in the literature regarding fear not necessarily due to COVID but due to other crises, other infectious diseases? Being able to make that comparison (recognizing that COVID-19 is of much larger proportion), might allow us to make some inference (even if not causal) on the magnitude of fear this time around.

Reply: We would like to thank you for this comment which we have duly acknowledged. We now have included Nepal specific information in the introduction section. Besides, COVID-19, we have also mentioned some other health system crisis faced by Nepal in the recent past.

You might want to consider using a multi-pronged approach for your recruitment methods (for next time). For example, people who are anxious or depressed or very fearful might be taking some time off social media - this limits the people who are responding to your survey to those who might not be feeling the extreme side of things. How else could you reach them? You can keep it web-based, but perhaps finding a listserv of emails from the professional organizations and sending them emails might be another opportunity to reach a wider range of people. (And people might be more willing to participate on a survey vs social media.)

Reply: Thank you for the suggestion which is helpful. We will consider the multi-pronged approach during future survey.

- How did you calculate your sample size? Why 475? What did the 475 participants represent in terms of representativeness of the country as a whole - were they working in urban/rural settings, were they older/younger, what type of facility? Who do these 475 participants represent? How did you classify “being involved in the COVID-19 response” - working directly with COVID-19 patients? Or working in a hospital in other departments? Working in research regarding the COVID-19 response?

Reply: We mainly focused our survey in COVID-19 dedicated hospitals which led to the greater representation of health workers working in central/province hospitals and private hospitals. These hospitals were mainly in urban settings, so was the concentration of COVID-19 cases during the time of the survey. These health workers were mainly involved in COVID-19 response either as frontline worker or were involved in management of pandemic.

- Something else to think about in terms of your population is that some might be triggered given their ongoing experience with COVID-19 and might not want to participate in this study - these limitations should be mentioned.

Reply: Thank you for the suggestion. We mentioned in the limitation as “Due to fear of COVID-19 pandemic, health care workers might not want to participate in the study.”

- For your logistic regression - it’s not necessary to rely on significance level (is there a reasoning for the 10%?) to create your model (in fact, it’s not recommended). Instead, you should use the literature to determine which variables should be included and accounted for. For example, has “awareness of government incentives” shown to determine fear? If so, based on your expertise and the literature, then yes, including it is great, if not, you might want to rethink its inclusion.

Reply: As many traditional levels such as p value of less than 0.05 fail to identify variables known to be important, we put variables which were significant at 0.1. Similar strategy has been suggested by Brusac Z et al in their article. Link to the article is <https://doi.org/10.1186/1751-0473-3-17>

Apart from the variables found on the literature review, we included some variables such as awareness of government incentives and incentives to health workers to match with the scenario of the government response. Any variables that did not show any association at 10% significance level were not further included in the model.

- In terms of health care workers, are community health care workers not included?

Reply: In this study, community health care workers were not included.

- You briefly mentioned this in the conclusion, but talking a bit more about the health system shortages in Nepal (lack of PPE, lack of widely available vaccines etc.) could also have contributed to the fear.

Reply: During the initial stage of COVID-19, there was wide shortage of PPE in the health facilities of Nepal. Vaccines were not available during the time of the survey.

- You also mentioned “effective strategies to reduce fear with a focus on nurses” - what are some potential recommendations (based on the literature) of strategies that can be helpful here?

Reply: Effective strategies to reduce fear with a focus on nurses could be support from the management especially with incentives, job rotation, and working hours, family and social support, psychological first aid, adequate personal protective equipment, and regular capacity building activities among others.
